# Similar exercise adherence and physical fitness outcomes are observed across distinct motivation profiles in older adults participating in a home-based structured exercise programme

**DOI:** 10.1186/s11556-026-00421-1

**Published:** 2026-06-18

**Authors:** Alexandra Munns, Jack Feron, Joan L. Duda, Sindre H. Fosstveit, Kelsey E. Joyce, Foyzul Rahman, Linda Wheeldon, Hilde Lohne-Seiler, Sveinung Berntsen, Jet Veldhuijzen van Zanten, Katrien Segaert, Samuel J. Lucas

**Affiliations:** 1 University of Birmingham, Birmingham, UK; 2 University of Agder, Kristiansand, Norway; 3 Loughborough University, Loughborough, UK; 4 Research Unit, Sorlandet Hospital, Kristiansand, Norway

**Keywords:** Ageing, Exercise adherence, Home-based exercise, Latent profile analysis, Autonomous motivation, Self-efficacy

## Abstract

**Background:**

Motivation and self-efficacy are well-established predictors of engagement and persistence within structured exercise programmes. Yet it remains unclear whether these psychological factors predict objective adherence metrics and associated fitness outcomes within home-based exercise programmes for older adults.

**Methods:**

This study adopted a person-centred approach to examine whether distinct psychological profiles predict objective adherence and fitness outcomes following a 26-week home-based exercise programme in 88 older adults (age = 67.2 ± 5.5 years). Objective adherence was defined as compliance with the prescribed exercise volume and quantified using cumulative metabolic equivalent minutes (MET-mins) derived from heart rate data reflecting session intensity and duration across the programme, as opposed to behavioural self-report measures. Psychological profiles were generated using latent profile analysis based on autonomous motivation, controlled motivation, and exercise self-efficacy.

**Results:**

Two profiles were identified: an autonomous and highly confident profile characterised by high self-efficacy and autonomous motivation, and a moderately autonomous and confident profile characterised by lower self-efficacy and autonomous motivation. Controlled motivation was similar between groups. Profiles did not differ in demographic or physiological characteristics at baseline. Adherence was high and comparable across the two profiles for all indices (e.g., cumulative MET-mins: 129 ± 66% vs. 116 ± 38%). Furthermore, both profiles demonstrated significant and comparable improvements in cardiorespiratory fitness (V̇O₂peak), handgrip strength, flexibility (chair sit-and-reach; back scratch), and mobility (8-foot up-and-go).

**Conclusions:**

Baseline differences in self-efficacy and autonomous motivation among older adults did not significantly predict adherence or fitness outcomes within a structured, well-supported home-based exercise programme. The programme incorporated consistent objective monitoring of exercise behaviour, along with regular guidance and feedback from the research team delivered through scheduled participant contact. This level of structured behavioural support may have reduced the influence of psychological profile membership on exercise adherence and fitness outcomes, resulting in comparable positive outcomes across participants. Future research should investigate whether motivation profiles become more predictive of long-term adherence and sustained fitness outcomes once external behavioural support is reduced or withdrawn.

**Trial registration:**

Open Science Framework: https://osf.io/6fqg7

## Background

Engaging in structured exercise can attenuate age-related decline in aerobic capacity and muscular strength, preserve functional ability, and contribute to the prevention and management of risk factors for major chronic non-communicable diseases [1–3]. Beyond its physiological effects, exercise can enhance psychological well-being, support independence, and improve quality of life in older adulthood [4, 5]. However, there is evidence that many older adults are insufficiently physically active to attain these potential health benefits; population data from England indicate that only around 63% of adults aged 55–74 and 43% of those aged 75 and over are classified as physically active [6]. Consistent with these national estimates, global surveillance data indicate that insufficient physical activity is widespread globally and increases markedly with age, with the highest prevalence observed among adults aged 60 years and older [7]. This limited engagement highlights a critical challenge in promoting healthy ageing, namely that not all individuals respond to, or engage with, exercise in the same way.

Even among older adults who participate in structured exercise programs, engagement and physical fitness- and function-related outcomes can vary considerably. The literature shows marked inter-individual variability in adherence to prescribed exercise and the resulting training responses [8–10]. Observed inter-individual variability in adherence and training-related responses may, in part, reflect non-modifiable influences such as age, baseline health, and genetic factors [11]. However, there is also evidence in older adults that the delivered exercise stimulus, particularly intensity and volume, is closely tied to training-induced adaptations [12–14]. Poor adherence can consequently reduce the therapeutic benefits of an exercise programme [14, 15]. It is therefore important that we examine modifiable factors that influence adherence and, in turn, variability in physical fitness and functional outcomes. In the present study, we examine psychological factors such as motivation and self-efficacy as key potential contributors [16, 17].

Theoretical frameworks can help to explain why older adults differ in their engagement with an exercise programme. Self-Determination Theory proposes that the quality of motivation, rather than its quantity, determines the likelihood of long-term behaviour [18]. Motivation is positioned on a continuum that ranges from amotivation, through controlled regulation, to autonomous regulation, reflecting the degree of volition that underpins behaviour [19, 20]. Controlled motivation refers to behaviour that is driven by external demands or internal pressures, such as exercising to avoid feelings of guilt or to meet the expectations of others, rather than from a sense of personal endorsement or enjoyment [19, 20]. Evidence demonstrates that older adults who report more autonomous reasons for exercise, such as engaging in physical activity because of the enjoyment or personal value, tend to show greater adherence and maintain higher levels of physical activity beyond structured interventions, whereas controlled forms of motivation are typically associated with short-term participation or early dropout [21–23].

In addition to motivational quality, Social Cognitive Theory complements this framework by identifying self-efficacy, defined as confidence in one’s ability to perform and sustain the behaviour, as a central determinant of behaviour [24]. In older adults, higher exercise self-efficacy has been shown to predict initiation, adherence, and the capacity to resume activity after a relapse [9, 25, 26]. Together, these frameworks suggest that the quality of motivation and self-efficacy can both influence individuals’ engagement with and adherence to structured exercise programmes.

Despite extensive research, three key conceptual and methodological limitations remain. Most studies have examined motivation and self-efficacy using variable-centred approaches that assess independent effects on exercise behaviour [23, 26, 27], overlooking how these constructs coexist within individuals to form distinct psychological profiles associated with engagement and training-related fitness and functional adaptations. In addition, few studies have examined whether motivation or self-efficacy are related to training-induced adaptations [28, 29]. Finally, adherence to exercise interventions in older adults is typically assessed using attendance or self-report rather than compliance with the prescribed exercise volume, and physiological indices of volume attainment are rarely incorporated [30–32]. Such behavioural metrics may therefore fail to identify individuals who attend sessions but do not achieve the intended training intensity, potentially contributing to the inconsistent associations reported between psychological predictors and exercise adherence [33–35]. Given that training-induced adaptations are influenced, in part, by the volume and intensity of exercise [12, 13], adherence measures reflecting compliance with the prescribed exercise volume are required to determine whether motivation and self-efficacy are related to variability in exercise-induced adaptations.

We address these gaps in the present study. We use a person-centred approach to examine how profiles of motivation and self-efficacy relate to compliance with the prescribed exercise volume and training-induced adaptations in older adults. Latent Profile Analysis (LPA) is used to identify distinct psychological profiles based on levels of autonomous motivation, controlled motivation, and self-efficacy. This approach extends beyond traditional variable-centred analyses by identifying naturally occurring combinations of psychological characteristics within individuals [36, 37]. In addition, adherence was assessed using indices of compliance with the prescribed exercise volume, alongside measures of physical fitness and function.

Our study had three key research objectives: first, to identify distinct psychological profiles among older adults about to undertake a 26-week home-based exercise programme using a person-centred analytic approach; second, to examine whether adherence indicators, including cumulative metabolic equivalent minutes (MET-mins), session number, duration, and heart rate-verified intensity, differ according to these profiles; and third, to assess whether these profiles predict differences in training responses to the home-based exercise programme, including changes in cardiorespiratory fitness, muscular strength, flexibility, mobility, and balance. This integrative approach provides novel insight into the interplay between motivation, self-efficacy, compliance with prescribed exercise volume, as well as associated fitness and functional adaptations in older adults.

## Methods

The data used in the present study were collected as part of a larger multi-site randomised controlled trial conducted in the United Kingdom and Norway that investigated the effects of a 26-week home-based exercise programme on a variety of outcome measures in healthy older adults. The present study includes novel data from secondary outcome measures, including motivation and self-efficacy, and addresses research questions that have not been examined in prior publications using data from this trial [38–41]. Reporting was conducted in accordance with the STROBE guidelines, and intervention details were described using the TIDieR checklist.

### Participants and demographics

The present analysis included older adults (aged 60–81 years) assigned to the exercise arm who completed a 26-week intervention and had complete psychological and adherence data (*n* = 88; 45 females, 43 males; 67.2 ± 5.5 years). Participants were free from current or prior serious health conditions, had no cognitive impairment, were non-smokers, and self-reported to not engage in ≥ 150 min/week of structured moderate-to-vigorous exercise, consistent with the screening criteria. Participant demographic characteristics are presented in the Results section (Table 1).

### 26-week home-based exercise programme

All participants completed a 26-week, home-based exercise programme, which has been described in detail previously [38]. Briefly, the exercise programme involved two high-intensity interval training (HIIT) sessions and one circuit-training session each week. Participants received ongoing guidance and follow-up from the research team throughout the programme. During the initial four-week familiarisation period, participants were contacted weekly, followed by monthly contact for the remainder of the programme. In addition to scheduled follow-ups, participants were able to contact the research team as needed throughout the programme. Researcher–participant contact involved regular communication to review training data, provide feedback on exercise intensity and progression, and address questions related to the programme. Follow-up was conducted through a combination of in-person meetings, email correspondence, and telephone or video calls, depending on practical considerations and participant preference. To support monitoring and adherence, participants were provided with a fitness watch (Polar Unite, Finland), a chest heart-rate monitor (Polar H9, Finland), and a logbook to record each session. Exercise intensity was guided by each participant’s percentage of peak heart rate (%HRpeak) established during the pre-intervention cardiorespiratory fitness test.

HIIT sessions consisted of alternating two-minute bouts of high-intensity exercise with active recovery periods. Participants were encouraged to reach > 80% HRpeak by the end of each high-intensity interval, with walking uphill, jogging, or running being the most chosen activities. Sessions began with five intervals per session and progressively increased to ten intervals. Circuit training sessions comprised six body-weight exercises (squats, high-knees, step-ups, press-ups, reverse lunges, and mountain climbers). Participants initially performed one set of each exercise during the familiarisation period, progressing to three sets of 45 s each from week five onwards. The aim was to complete as many repetitions as possible within each set and achieve > 80% HRpeak by the end of each exercise bout [39]. See Fosstveit et al. for full details [38].

### Psychological measures

To address Objective 1, we classified participants into distinct psychological profiles based on their shared patterns of autonomous motivation, controlled motivation, and self-efficacy. Motivation for exercise was assessed using an adapted version of the Behavioural Regulation in Exercise Questionnaire-2 (BREQ-2) [42]. Autonomous motivation was calculated as the mean of the intrinsic motivation and identified regulation subscales, whereas controlled motivation was calculated as the mean of the introjected and external regulation subscales [21]. Scores for both autonomous and controlled motivation ranged from 0 to 4. Higher scores on each construct indicate a stronger tendency to regulate exercise behaviour through that respective motivational orientation. Internal consistency reliability was examined separately for the UK and Norway samples. Cronbach’s alpha values indicated good reliability for autonomous motivation across both countries (UK: α = 0.88; Norway: α = 0.78). Controlled motivation demonstrated acceptable reliability (UK: α = 0.68; Norway: α = 0.75), supporting the cross-cultural applicability of the BREQ-2 in this sample.

Self-efficacy was measured using the Barriers Self-Efficacy Scale (BARSE) [43]. Participants rated their confidence (0–100%) in exercising three times a week despite common barriers (e.g., fatigue, poor weather). A composite score was calculated as the mean of all 13 questionnaire items, with higher values reflecting greater perceived confidence in overcoming barriers to exercise participation. The BARSE has demonstrated strong reliability and predictive validity for physical activity behaviour in older adults [44, 45]. Internal consistency was likewise tested separately by country to verify the reliability of measurement across samples. Cronbach’s alpha indicated excellent reliability for the BARSE (UK: α = 0.93; Norway: α = 0.94), confirming that the measure consistently captured perceived exercise self-efficacy across countries.

### Exercise programme adherence

To address Objective 2, we examined whether adherence to the exercise programme differed between profiles. Adherence was operationalised as compliance with the prescribed exercise volume and quantified using heart-rate data measured via a chest-worn monitor and recorded by a fitness watch, from which session number, duration, and heart-rate responses (mean, peak, and time spent within target zones) were derived. Adherence metrics are expressed as the percentage of the prescribed exercise volume completed. The primary adherence metric was the percentage of cumulative metabolic equivalent minutes (MET-mins) achieved across the programme [46]. MET-mins for each session were calculated by multiplying the recorded session duration by the MET value corresponding to the mean percentage of peak heart rate [35]. Cumulative MET-mins for each participant were calculated by summing the MET-mins across all completed sessions. In addition to this primary metric, session number, session duration, and session intensity (defined as minutes per session > 80% of peak heart rate) were examined as complementary indicators to characterise adherence behaviour. Full methodological details of adherence calculations are reported in Feron et al. [39].

### Physical fitness and functional outcome measures

To address Objective 3, we examined whether changes in physical fitness and functional outcomes across the exercise programme differed between the identified psychological profiles. Assessments were conducted at baseline and post-intervention using a battery of validated measures of cardiorespiratory fitness and physical function.

#### Cardiorespiratory fitness

Peak oxygen uptake (V̇O₂peak) was assessed using a modified Balke treadmill protocol to volitional exhaustion, as described previously [38, 39]. Briefly, respiratory gases were measured continuously via facemask (Hans Rudolph, USA) and a Vyntus CPX metabolic cart (Vyaire, Illinois, USA). Participants completed 4-minute walking stages (3.8 km·h⁻¹) separated by 1-minute rest periods. Treadmill incline increased at each stage (4–20%) until lactate threshold was reached. Following lactate threshold, treadmill speed and/or gradient were increased every 1 min until volitional exhaustion. Heart rate was monitored throughout (Norway: Polar H9, UK:12-lead ECG) and ratings of perceived exertion [47] were recorded after each stage. V̇O₂peak was calculated as the mean of the two highest consecutive 30-second oxygen uptake values.

#### Physical function

Handgrip strength was measured using a hand-held dynamometer, with a Takei dynamometer (model 5001, Takei Scientific Instruments, Japan) used in the UK and a hydraulic hand dynamometer (model SH5001, Saehan, Korea) used in Norway [48]. The dynamometer was adjusted for the grip size of the dominant hand. Participants stood upright with arms by their sides, squeezing maximally while maintaining elbow extension and limiting shoulder movement. Three attempts were performed, and the highest value (kg) was recorded.

Lower-body strength was assessed with the 30-second chair-stand test [49]. Participants sat upright in a chair with both feet flat on the floor, shoulder width apart, and hands crossed over their chest. Participants had one attempt to fully stand up (knees fully extended) and fully sit down as many times as possible in 30 s.

Flexibility was measured using the chair sit-and-reach and back scratch tests [49]. For the chair sit-and-reach, participants sat at the edge of a chair, extended one leg forward with the heel on the floor, and toes pointed upward. Participants interlaced their fingers and reached towards their toes, getting as close to or as far past their toes as they could. The distance (cm) reached beyond (positive) or short of (negative) the toes was measured with a ruler. The best score from two attempts was used.

For the back scratch test, participants stood upright and placed one hand over the same shoulder, reaching down the back as far as possible, while the opposite hand reached up the middle of the back from below, attempting to touch or overlap the middle fingers. The distance (cm) between the middle fingertips was measured with a ruler, with overlap recorded as a positive score and a gap as a negative score. The test was performed on both sides (right and left), and the best score from the two trials was recorded.

Mobility was evaluated using the 8-foot up-and-go test [49]. Participants sat upright on a chair with both feet on the floor and hands on their knees. Participants were asked to walk out to and around a cone placed 8 feet away and return to their seat as fast as possible without running. Two trials were completed, and the fastest time(s) were recorded.

Balance was assessed using the one-leg stance test. Participants stood on one leg of their choice, placing the heel of the non-standing leg against the medial knee of the standing leg. Arms were unrestricted, and participants attempted to maintain balance for up to 60 s. After one brief practice, two recorded trials were completed, and the best time (s) were recorded.

Higher scores indicated better physiological function across all domains, except for the 8-foot up-and-go test, where lower times reflected superior performance.

### Latent profile analysis: establishing distinct psychological profiles (Objective 1)

Latent profile analysis (LPA) was conducted in R (Version 4.4.3) [50] to identify subgroups of older adults based on psychological characteristics. Three indicators were included: autonomous motivation, controlled motivation, and self-efficacy. Models specifying one, two, and three profiles were estimated. Model selection was guided by statistical and theoretical considerations, including the Akaike Information Criterion (AIC) [51], the Bayesian Information Criterion (BIC) [52], the sample-size adjusted BIC (SABIC), and entropy. Lower AIC, BIC, and SABIC values, in combination with higher entropy, were taken to indicate superior model fit and clearer classification. The final two-profile model was selected as optimal, balancing statistical fit, parsimony, and theoretical interpretability.

All subsequent analyses were conducted using IBM SPSS Statistics, Version 29.0 (IBM Corp., Armonk, NY). To characterise the latent profiles, differences in autonomous motivation, controlled motivation, and self-efficacy were examined across profiles. Demographic characteristics (age, sex, country) and baseline physical fitness and functional outcomes (V̇O₂peak, handgrip strength, 30-second chair stand, chair sit-and-reach, back scratch, balance test, and 8-foot up-and-go) were compared between profiles using one-way ANOVAs for continuous variables and chi-square (χ²) tests for categorical variables.

### Statistical analyses: differences between profiles in adherence (objective 2) and physical fitness & function (Objective 3)

To address Research Objective 2, differences in adherence to the exercise programme between latent profiles were examined using a series of one-way ANOVAs, with latent profile (moderately autonomous and confident vs. autonomous and highly confident) entered as the independent variable. Dependent variables comprised the four adherence indices described previously: cumulative metabolic equivalent minutes (MET-mins), session intensity (minutes > 80% HRₘₐₓ), session number, and session duration.

To address Research Objective 3, 2 (Time: pre, post) × 2 (Latent Profile: moderately autonomous and confident vs. autonomous and highly confident) repeated-measures ANOVAs were conducted for each physical fitness and functional outcome, including peak oxygen uptake (V̇O₂peak), handgrip strength, 30-second chair stand, chair sit-and-reach, back scratch, balance, and the 8-foot up-and-go test. Where appropriate, significant effects were followed up with pairwise comparisons. Effect sizes are reported as partial eta squared (η²ₚ), and statistical significance was set at *p* < .05.

## Results

Participant characteristics across the total sample and latent profiles are presented in Table 1.

**Table 1:** Participant Characteristics

Variable	Total	Moderately Autonomous & Confident	Autonomous & Highly Confident
n	88	49 (55.7%)	39 (44.3%)
Age (years)	67.2 ± 5.5	66.6 ± 5.1	67.9 ± 6.1
Body mass (kg)	79.9 ± 12.5	79.6 ± 11.6	80.3 ± 13.9
Height (cm)	172 ± 10	171 ± 8.4	172 ± 11.4
Sex			
Female	45 (51.4%)	25 (51%)	20 (51.3%)
Male	43 (48.9%)	24 (49%)	19 (48.7%)
Country			
UK	35 (39.8%)	20 (40.8%)	15 (38.5%)
Norway	53 (60.2%)	29 (59.2%)	24 (61.5%)
Highest education level reached			
Compulsory	11 (12.5%)	5 (10.2%)	6 (15.4%)
Further	23 (26.1%)	14 (28.6%)	9 (23.1%)
Undergraduate	26 (29.6%)	13 (26.5%)	13 (33.3%)
Postgraduate	28 (31.8%)	17 (34.7%)	11 (28.2%)

No significant differences in demographic characteristics were observed between latent profiles (all *p* > .05).

### Latent profile analysis: identification of distinct psychological profiles in older Adults (Objective 1)

Latent profile analysis identified two distinct psychological profiles among older adults participating in the exercise programme. Fit statistics for the one-, two-, and three-class solutions are presented in Table 2. Both the Akaike Information Criterion (AIC) and the sample-size adjusted BIC (SABIC) decreased as additional classes were specified, indicating incremental improvements in model fit. Entropy values exceeded 0.70 for the two- and three-class solutions, suggesting adequate classification quality. Although the three-class model demonstrated slightly lower AIC and SABIC values compared to the two-class solution, it produced a small subgroup that was less stable, given the modest sample size, and offered limited theoretical distinction. Therefore, the two-class solution was selected as the most parsimonious and interpretable representation of the data.

**Table 2:** Fit indices for latent profile models (*N* = 88)

Classes	LogLik	AIC	BIC	SABIC	Entropy
1	-609	1236	1258	1230	1.000
2	-580	1198	1246	1186	0.745
3	-569	1196	1268	1177	0.836

**Fig. 1:**
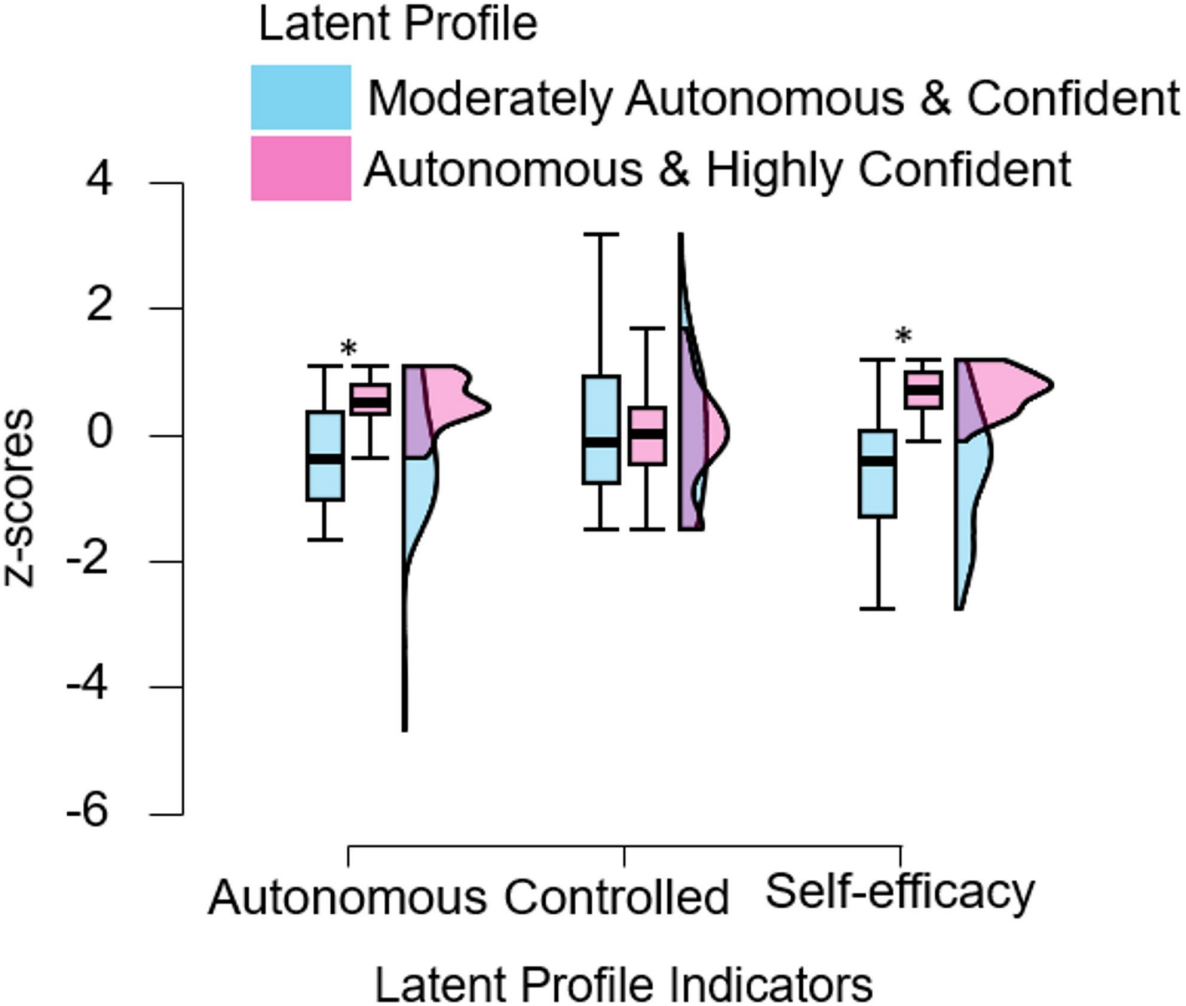
Standardised psychological indicators characterising the two latent profiles. Note. Values represent standardised z-scores for autonomous motivation, controlled motivation, and self-efficacy. Boxplots represent medians and interquartile ranges, with whiskers indicating minimum and maximum values. Overlaid density distributions illustrate the distribution of scores. * indicates between-profile differences at *p* < .001

To characterise the latent profiles, differences in controlled motivation, autonomous motivation, and self-efficacy were examined between profiles. The two profiles reported similarly low levels of controlled motivation, F(1, 86) = 0.78, *p* = .38, η²ₚ = 0.01 (moderately autonomous and confident: 1.15 ± 0.81; autonomous and highly confident: 1.02 ± 0.63). In contrast, autonomous motivation was higher in the autonomous and highly confident profile than in the moderately autonomous and confident profile, F(1, 86) = 25.40, *p* < .001, η²ₚ = 0.23 (3.60 ± 0.29 vs. 2.98 ± 0.80). Similarly, differences were observed for self-efficacy, F(1, 86) = 66.40, *p* < .001, η²ₚ = 0.44, with substantially higher values in the autonomous and highly confident profile (87.46 ± 8.38) than in the moderately autonomous and confident profile (56.33 ± 25.32). The moderately autonomous and confident profile also demonstrated greater variability across the psychological indicators. These patterns are illustrated in the standardised z-score profiles shown in Fig. 1.

### Adherence does not differ between the autonomous and highly confident and moderately autonomous and confident profiles (Objective 2)

**Fig. 2:**
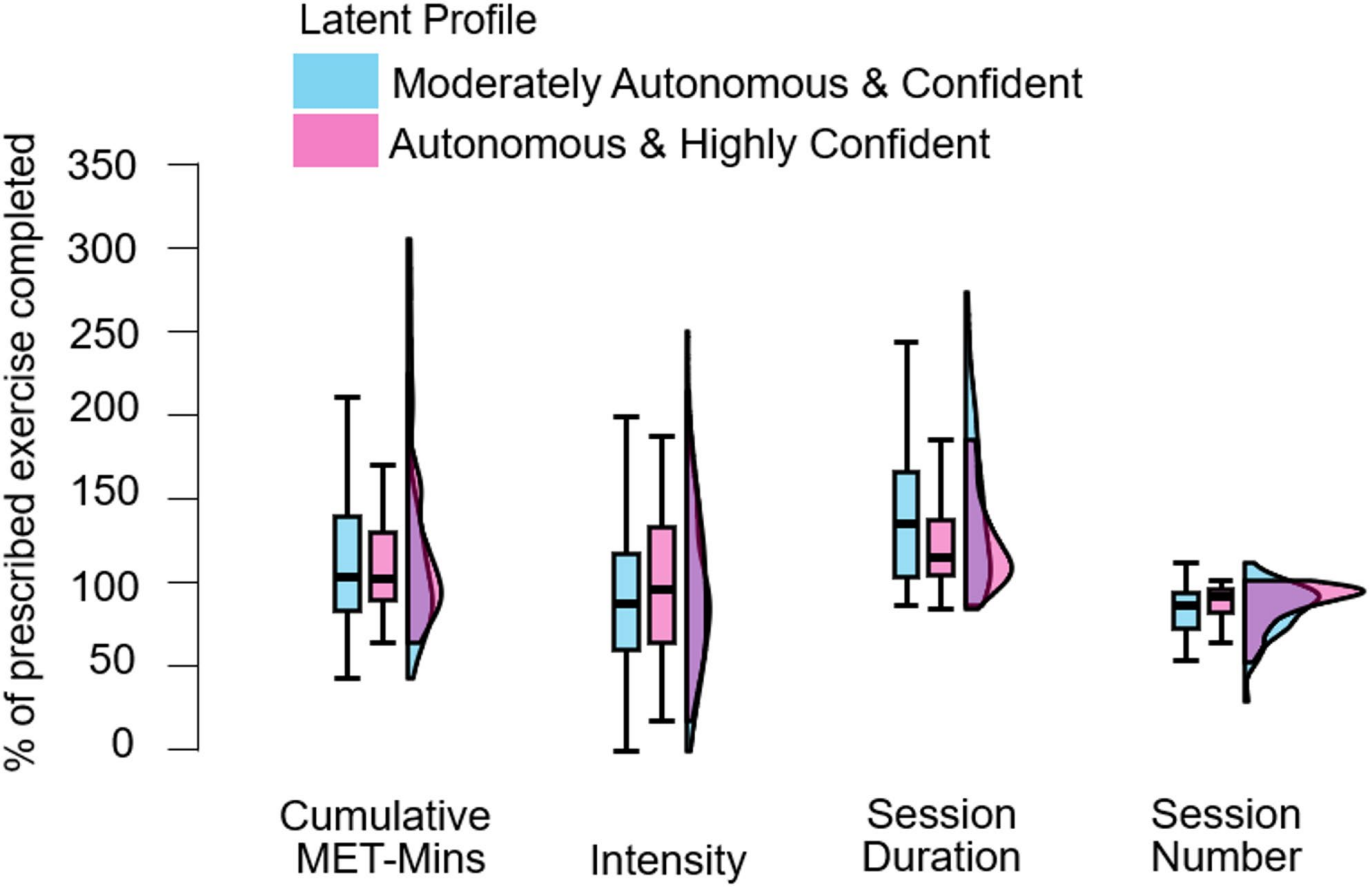
Exercise adherence metrics across latent profiles. Note. Boxplots represent median and interquartile range, with whiskers indicating minimum and maximum values. Violin plots depict the distribution of values for each latent profile. Values are expressed as a percentage of the prescribed exercise dose. MET: metabolic equivalent

Descriptive statistics for adherence outcomes are presented in Table 3; Fig. 2. Both profiles demonstrated high engagement with the intervention across all adherence indices. No significant differences were observed between the autonomous and highly confident and moderately autonomous and confident profiles on any adherence metric (all *p* > .05).

**Table 3:** Prescribed exercise volume and percentage adherence outcomes across latent profiles

Adherence Metric		% Adherence to Prescribed Volume	
	Prescribed Exercise Volume	Moderately Autonomous & Confident	Autonomous & Highly Confident
Cumulative MET-Mins	8567 MET-mins	128.6 ± 66.1	116.2 ± 37.9
Session Number	66 sessions	84.1 ± 15.8	88.9 ± 12.5
Session Duration	29.5 min/session	145.8 ± 46.9	125.9 ± 26.8
Intensity (> 80%HRpeak)	10.5 min/session	88.6 ± 48.5	108.8 ± 47.5

Values are presented as mean ± standard deviation percentage adherence relative to the prescribed exercise volume. Adherence metrics were calculated using post-familiarisation data only (i.e., Weeks 5–26). Prescribed volume for session duration and intensity represent average target values calculated across the 66 prescribed exercise sessions. Intensity was defined as minutes per session completed at > 80% of peak heart rate (HRpeak)

*MET* metabolic equivalent

### Physical fitness and functional outcomes do not differ between the autonomous and highly confident and moderately autonomous and confident profiles (Objective 3)

Repeated-measures ANOVAs examined exercise programme-induced changes in physical fitness and functional outcomes by latent profile. Results are summarised in Table 4. Apart from balance (*p* = .053), significant main effects of time were observed for V̇O₂peak, handgrip strength, chair stand performance, chair sit-and-reach, back scratch, and the 8-foot up-and-go, indicating improvements from pre- to post-intervention (all *p* < .05). There were no significant main effects of profile, and no significant time × profile interactions (see Table 4).

**Table 4:** Repeated-measures ANOVA results for fitness and functional outcomes by latent profile. Listed are mean values (± standard deviation) for each outcome variable pre- and post-intervention, with F-values (test statistics) and η²ₚ values (effect sizes representing the proportion of variance explained) for the effects of time, latent profile (moderately autonomous and confident vs. autonomous and highly confident), and their interaction. Significance is denoted by * for *p* < .05 and *** for *p* < .001

Outcome	Moderately Autonomous & Confident		Autonomous & Highly Confident		F(df)	Time		Profile		Time × Profile	
	pre	post	pre	post		F	η²ₚ	F	η²ₚ	F	η²ₚ
V̇O₂peak (ml/kg/min)	27.8 ± 5.3	29.3 ± 5.4	27.6 ± 5.4	29.1 ± 5.3	(1, 75)	33.56***	0.309	0.02	< 0.001	< 0.01	< 0.001
Handgrip Strength (kg)	34.6 ± 10.9	36.6 ± 10.8	32.5 ± 10.6	35.2 ± 10.5	(1, 80)	49.04***	0.38	0.45	0.006	0.06	0.001
Chair Stand (reps)	14.39 ± 3.3	16.7 ± 3.6	14 ± 2.2	16.7 ± 2.5	(1, 81)	143.83***	0.64	0.23	0.003	1.15	0.014
Chair Sit-and-Reach (cm)	1.2 ± 12.7	3.6 ± 12.2	1.6 ± 10.1	2.7 ± 11.2	(1, 82)	6.55*	0.074	0.05	0.001	0.27	0.003
Back Scratch (cm)	-10.2 ± 11.1	-7.9 ± 11.2	-8.3 ± 10.2	-5 ± 11.5	(1, 80)	34.27***	0.3	0.94	0.012	0.15	0.002
8-Foot Up & Go (s)	5.3 ± 0.9	4.9 ± 0.8	5.4 ± 0.7	4.9 ± 0.5	(1, 81)	82.56***	0.505	0.01	< 0.001	0.17	0.002
Balance (score)	34.5 ± 22.8	37.7 ± 20.8	28.2 ± 22.4	32.3 ± 21.7	(1, 82)	3.84	0.045	1.26	0.015	0.08	0.001

Values are presented as mean ± standard deviation

*η²ₚ * partial eta squared, *V̇̇O₂peak*   peak oxygen uptake

Significant values are shown in bold; * *p* < .05*; *** *p* < .001

## Discussion

This study examined whether psychological profiles were associated with objectively assessed adherence and improvements in physical fitness and function following a 26-week home-based exercise programme in older adults. Using person-centred latent profile analysis (Objective 1), generally healthy older adults were classified into two distinct psychological profiles: an Autonomous and Highly Confident profile and a Moderately Autonomous and Confident profile. Despite substantial differences in autonomous motivation and self-efficacy between profiles, adherence to the prescribed exercise programme (Objective 2) and training-related outcomes (Objective 3) were comparable, indicating that both profiles responded similarly to the structured exercise programme.

Contrary to what might be expected based on differences in levels of autonomous motivation and self-efficacy, the Autonomous and Highly Confident profile did not exhibit higher adherence than the Moderately Autonomous and Confident profile, despite both profiles reflecting generally favourable motivational characteristics. Previous systematic reviews indicate that adherence to home-based exercise interventions in older adults varies considerably. For example, Chaabene et al. reported a mean compliance rate of roughly 70%, although only 50% of included studies provided adherence data [53]. Liang et al. identified adherence estimates ranging from 30% to 96% across 90% of studies that reported adherence [54], while Geraedts et al. observed adherence rates between 32.1% and 91% across 69% of included studies [55]. The uniformly high adherence observed across both profiles in the present study may have limited variability, reducing the ability to detect between-profile differences. This may reflect generally high autonomous motivation across the sample, with participants in both profiles demonstrating consistent compliance with the prescribed exercise volume. Although differences in intervention modality and intensity limit direct comparison, adherence levels in the present study fall toward the upper end of the ranges reported in the existing literature.

Interpretation of adherence findings across studies is further constrained by substantial methodological heterogeneity. Adherence is often inconsistently defined and assessed using self-report methods such as training logs, telephone check-ins, or retrospective questionnaires [53, 54]. In contrast, the present study employed an objective definition of adherence, operationalised as compliance with the prescribed exercise volume, representing a methodological strength that enhances the precision and validity of adherence assessment [56, 57]. Adherence was indexed using heart-rate-guided intensity thresholds, confirming that exercise sessions were completed and performed at the prescribed intensity. This approach reduces recall and social desirability biases commonly associated with self-reported adherence in home-based interventions [58, 59], providing a robust basis for examining associations between adherence and subsequent outcome measures [60].

The exercise programme was implemented with several contextual features. Although sessions were completed independently at home, the programme incorporated regular researcher contact, personalised feedback, structured and progressive exercise prescriptions, and wearable monitoring during exercise. These components align with behaviour change techniques known to support exercise engagement in older adults, particularly self-monitoring and personalised feedback [61–63]. Additionally, regular researcher contact may have provided external support and accountability, consistent with approaches used to promote longer-term exercise adherence in older populations [64]. Systematic reviews similarly report that home-based interventions offering ongoing monitoring and feedback achieve higher and more stable adherence than minimally supported or unsupervised formats [55, 65, 66]. These features likely contributed to uniformly high engagement across participants, thereby reducing behavioural variability and limiting the discernibility of adherence differences between latent profiles.

Post-intervention questionnaire responses provided further contextual insight into participants’ experiences of the intervention. Overall, 94% of participants included in the present study completed the post-intervention questionnaire, of whom 87% reported enjoying the overall six-month exercise programme either ‘very much’ or ‘extremely’. Additionally, 58% of participants either ‘slightly agreed’ or ‘strongly agreed’ that they would have struggled to complete the programme without regular researcher contact, and 57% similarly indicated that they would have completed fewer exercise sessions in the absence of this support. Although these measures were not primary outcomes of the present analysis, they may help contextualise the consistently high levels of programme engagement observed across participants, as well as the role of regular researcher contact and structured support throughout the intervention.

In this sample, participants with higher autonomous motivation and self-efficacy did not exhibit greater training-related adaptations, as both profiles showed comparable improvements in cardiorespiratory fitness and physical function. This pattern aligns with the adherence data, as similar training exposure across profiles would be expected to result in comparable adaptations. This finding contributes to a limited and methodologically diverse literature with equivocal evidence regarding whether psychological factors prospectively predict exercise-induced changes in physical fitness and function. Among older cardiac patients participating in an eight-week supervised rehabilitation programme, higher baseline autonomous motivation predicted greater improvements in V̇O₂peak [28]. In contrast, a 16-week resistance-training intervention in middle-aged adults found that baseline self-efficacy and behavioural regulation did not predict overall strength improvements [29]. These divergent findings likely reflect key methodological differences. Notably, adherence was assessed only as programme completion or not reported in these studies, limiting insight into whether differences in training exposure contributed to the observed training-induced adaptations. Each study examined different psychological constructs in isolation, used variable-centred analyses, and was conducted in distinct training contexts and populations. By contrast, the present study used a person-centred approach, where participants were grouped based on a combination of their levels of autonomous motivation and self-efficacy, allowing examination of how the co-occurrence of these characteristics was associated with adherence [67, 68]. Taken together, these findings suggest that relationships between psychological attributes and training-induced adaptations may be more nuanced than single-construct models indicate, particularly when training volume is closely standardised and objectively verified based on physiological data rather than self-reported participation.

Our findings contribute to a wider literature on Self-Determination Theory and Social Cognitive Theory [19, 24]. Both frameworks propose that autonomous motivation and self-efficacy support behavioural processes such as intention formation, persistence, and the self-regulation of effort that facilitate sustained engagement in exercise participation. Empirical evidence in older adults similarly shows that higher autonomous motivation and self-efficacy are associated with greater participation in exercise [26, 69–71]. In parallel, studies show that adherence to training intensity and volume predicts the extent of exercise-induced adaptations achieved [12–14]. On this basis, it was anticipated that the profile characterised by higher autonomous motivation and self-efficacy would demonstrate greater behavioural engagement, leading to more pronounced training-related improvements. However, motivation and self-efficacy levels in the Moderately Autonomous and Confident profile remained within a moderate-to-high range. Importantly, the self-efficacy measure used in this study captured confidence to continue exercising despite common barriers, rather than confidence to exercise at a prescribed intensity. From a Social Cognitive Theory perspective, such barrier-related self-efficacy may be more strongly related to persistence and attendance than to achieving specific training intensities [24]. Voluntary enrolment in a structured exercise programme may therefore indicate that participants across both profiles possessed sufficient task-specific self-efficacy to sustain participation. Under these conditions, observed differences in self-efficacy may have been insufficient to produce differential adherence or fitness and functional outcomes.

The results indicate that when both profiles begin from relatively strong motivational perspectives and receive substantial structural support, adherence to the prescribed training stimulus becomes comparable across profiles, with most participants achieving a training volume sufficient to elicit meaningful training-related improvements. Under these conditions, observed changes are likely to reflect the characteristics of the exercise stimulus and normal inter-individual biological variation, consistent with evidence that training volume and intensity are primary determinants of improvements in physical fitness and function [13, 35]. From this perspective, comparable adherence to the prescribed training stimulus across profiles may have limited the extent to which autonomous motivation and self-efficacy could differentially influence fitness and functional outcomes. It should also be acknowledged that this level of structural support was delivered within a research setting and may have introduced additional influences on engagement and adherence beyond the exercise programme itself. Accordingly, caution is warranted when applying these findings to real-world contexts, where access to comparable levels of support may be more limited.

These findings provide valuable insights into adherence to the prescribed training stimulus and the effectiveness of the home-based exercise programme during the active delivery period. However, continuation of exercise behaviour was not assessed beyond this period, and it therefore remains unclear whether motivational and self-efficacy profiles differentially predict the maintenance of exercise behaviour once the programme has ended. Future research should incorporate longer-term follow-up assessments to examine whether these psychological profiles are associated with the maintenance of exercise behaviour over time, when individuals rely more heavily on self-regulation [11, 16, 17]. Furthermore, future studies should investigate whether these profiles play distinct roles across different delivery contexts. Specifically, it remains unclear whether similar profile effects would be observed in less structured or minimally supported settings, where adherence and training intensity are not directly monitored. Motivation and behaviour-change research suggests that autonomous motivation and self-efficacy may be particularly important in such real-world, unsupervised contexts where external structure and support are limited [21, 71].

The current results also have implications for the design and delivery of home-based exercise programmes for older adults. Specifically, within a structured and well-supported delivery context, individuals with differing motivational and self-efficacy profiles demonstrated comparable adherence and achieved similar improvements in physical fitness and function. Programme-level features such as clear exercise prescriptions, objective self-monitoring, and the provision of regular feedback have been widely identified as relevant components of effective home-based exercise programmes for older adults and may help to standardise training exposure across participants [61, 65, 72]. Nevertheless, evidence suggests that engagement with technology-supported self-monitoring is context dependent, underscoring the need to establish whether comparable outcomes are observed in less structured or minimally supported real-world settings [72, 73].

The sample was modest in size and comprised cognitively healthy, motivated, and physically healthy older adults, which limits generalisability to the wider older adult population. Future studies should aim to recruit larger and more diverse samples, including individuals with lower baseline motivation, greater health burden, and a broader range of cognitive and functional abilities. The identification of two psychologically distinct profiles within this relatively homogeneous group indicates that meaningful differences in autonomous motivation and self-efficacy can exist even among engaged and functionally independent older adults [67, 74]. This supports the value of person-centred analytical approaches, such as latent profile analysis, for capturing naturally occurring configurations of psychological characteristics that may be obscured using traditional variable-centred methods [37, 75].

## Conclusions

This study provides novel insight into how motivational and self-efficacy profiles in healthy older adults relate to objectively assessed exercise adherence and training-related improvements in physical fitness and function during a 26-week home-based exercise programme. Using a person-centred latent profile analysis, two psychologically distinct profiles were identified based on autonomous motivation and self-efficacy. Despite these differences, both profiles demonstrated comparable adherence and achieved similar improvements in physical fitness and function. Adherence levels were uniformly high, which may have been caused by the structured nature and/or relatively high efficacy and motivation levels in both profiles. Under these conditions, baseline differences in autonomous motivation and self-efficacy did not influence short-term responses in cardiorespiratory fitness or physical functions, though this should not be taken to suggest that such psychological factors lack broader relevance. Future research should examine their influence in less structured, real-world settings and during longer-term follow-up, where external support is reduced, and individuals must regulate their behaviour more independently. By combining person-centred psychological profiling with objective assessment of training volume, this study advances understanding of how psychological factors interface with exercise exposure and adaptation in later life.

## Data Availability

The datasets supporting the conclusions of this article are available in the Open Science Framework repository: (https://osf.io/d7aw2). The study was preregistered on the Open Science Framework: (https:/osf.io/6fqg7). Transparent documentation of changes pertaining to the present manuscript is available at (https:/osf.io/dtw69).

## Abbreviations

AIC: Akaike Information Criterion
ANOVA: Analysis of variance
BARSE: Barriers Self-Efficacy Scale
BIC: Bayesian Information Criterion
BREQ-2: Behavioural Regulation in Exercise Questionnaire-2
ECG: Electrocardiogram
FAB: Fitness, Ageing and Bilingualism
HIIT: High-intensity interval training
HRpeak: Peak heart rate
LPA: Latent profile analysis
MET: Metabolic equivalent
MET-mins: Metabolic equivalent minutes
SABIC: Sample-size adjusted Bayesian Information Criterion
SPSS: Statistical Package for the Social Sciences
V̇O₂peak: Peak oxygen uptake

## References

[1] Fernandez-Gamez B, Solis-Urra P, Olvera-Rojas M, Molina-Hidalgo C, Fernández-Ortega JA, Lara CP, et al. Resistance exercise program in cognitively normal older adults: CERT-based exercise protocol of the AGUEDA randomized controlled trial. J Nutr Health Aging. 2023;27(10):885–93. 37960912 10.1007/s12603-023-1982-1 PMC12880457

[2] Buriticá-Marín ED, Daza-Arana JE, Jaramillo-Losada J, Riascos-Zuñiga AR, Ordoñez-Mora LT. Effects of a physical exercise program on the physical capacities of older adults: a quasi-experimental study. Clin Interv Aging. 2023;18:273–82. 36851976 10.2147/CIA.S388052 PMC9960785

[3] Fairag M, Alzahrani SA, Alshehri N, Alamoudi AO, Alkheriji Y, Alzahrani OA, et al. Exercise as a therapeutic intervention for chronic disease management: a comprehensive review. Cureus. 2024;16(11):e74165. 39712722 10.7759/cureus.74165 PMC11662992

[4] Benlidayı İC. The effects of exercising on psychological well-being in older adults. Anti-Aging East Eur. 2023;2(1):36–41.

[5] Figueira HA, Figueira OA, Corradi-Perini C, Martínez-Rodríguez A, Figueira AA, da Silva CRL, et al. A descriptive analytical study on physical activity and quality of life in sustainable aging. Sustainability. 2021;13(11):5968.

[6] Sport England. Active Lives Adult Survey November 2023–24. London: Sport England. 2025. Available from: https://sportengland.org. Accessed 15 Jan 2025.

[7] Strain T, Flaxman S, Guthold R, Semenova E, Cowan M, Riley LM, et al. National, regional, and global trends in insufficient physical activity among adults from 2000 to 2022: a pooled analysis of 507 population-based surveys with 5.7 million participants. Lancet Glob Health. 2024;12(8):e1232–43. 38942042 10.1016/S2214-109X(24)00150-5 PMC11254784

[8] Cano-Montoya J, Hurtado N, Vergara CN, Vargas SB, Rojas-Vargas M, Martínez-Huenchullán S, et al. Interindividual variability response to resistance and high-intensity interval training on blood pressure reduction in hypertensive older adults. J Cardiovasc Dev Dis. 2025;12(1):30. 39852308 10.3390/jcdd12010030 PMC11765815

[9] Shaw JF, Pilon S, Vierula M, McIsaac DI. Predictors of adherence to prescribed exercise programs for older adults with medical or surgical indications for exercise: a systematic review. Syst Rev. 2022;11(1):84. 35488307 10.1186/s13643-022-01966-9 PMC9052492

[10] Di Lorito C, Long A, Bryan A, Harwood RH, Gladman JRF, Schneider S, et al. Exercise interventions for older adults: a systematic review of meta-analyses. J Sport Health Sci. 2021;10(1):29–47. 32525097 10.1016/j.jshs.2020.06.003 PMC7858023

[11] Noone J, Mucinski JM, DeLany JP, Sparks LM, Goodpaster BH. Understanding the variation in exercise responses to guide personalized physical activity prescriptions. Cell Metab. 2024;36(4):733–48. 10.1016/j.cmet.2023.12.025 38262420

[12] Oliveira A, Fidalgo A, Farinatti P, Monteiro W. Effects of high-intensity interval and continuous moderate aerobic training on fitness and health markers of older adults: a systematic review and meta-analysis. Arch Gerontol Geriatr. 2024;124:105451. 38718488 10.1016/j.archger.2024.105451

[13] Lai X, Zhu H, Wu Z, Chen B, Jia Q, Du H, et al. Dose-response effects of resistance training on physical function in frail older Chinese adults: a randomized controlled trial. J Cachexia Sarcopenia Muscle. 2023;14(6):2824–34. 37875291 10.1002/jcsm.13359 PMC10751415

[14] Nagata CA, Garcia PA, Hamu TCDS, Caetano MBD, Costa RR, Leal JC, et al. Are dose-response relationships of resistance training reliable to improve functional performance in frail and pre-frail older adults? A systematic review with meta-analysis and meta-regression of randomized controlled trials. Ageing Res Rev. 2023;91:102079. 37774931 10.1016/j.arr.2023.102079

[15] Sáez de Asteasu ML, Martínez-Velilla N, Zambom-Ferraresi F, Galbete A, Ramírez-Vélez R, Cadore EL, et al. Dose-response relationship between exercise duration and enhanced function and cognition in acutely hospitalized older adults: a secondary analysis of a randomized clinical trial. Innov Aging. 2024;8(6):igae050. 38939651 10.1093/geroni/igae053 PMC11208931

[16] O’Neil-Pirozzi TM, Cattaneo G, Solana-Sánchez J, Gomes-Osman J, Pascual-Leone A. The importance of motivation to older adult physical and cognitive exercise program development, initiation, and adherence. Front Aging. 2022;3:773944. 35821853 10.3389/fragi.2022.773944 PMC9261340

[17] Kim HR, Woo SH, Seo JP, So WY, Bae JS. Satisfaction with the exercise program and successful aging among older adults who exercise regularly: the multiple mediation of physical self-efficacy and exercise adherence. Healthc (Basel). 2024;12(20):2054. 10.3390/healthcare12202054 PMC11506852 39451468

[18] Ryan RM, Deci EL. Self-determination theory and the facilitation of intrinsic motivation, social development, and well-being. Am Psychol. 2000;55(1):68–78. 11392867 10.1037//0003-066x.55.1.68

[19] Ryan RM, Deci EL. Self-determination theory: basic psychological needs in motivation, development, and wellness. New York (NY): Guilford Press; 2017.

[20] Hagger MS, Hardcastle SJ, Chater A, Mallett C, Pal S, Chatzisarantis NLD. Autonomous and controlled motivational regulations for multiple health-related behaviors: between- and within-participants analyses. Health Psychol Behav Med. 2014;2(1):565–601. 25750803 10.1080/21642850.2014.912945 PMC4346087

[21] Teixeira PJ, Carraça EV, Markland D, Silva MN, Ryan RM. Exercise, physical activity, and self-determination theory: a systematic review. Int J Behav Nutr Phys Act. 2012;9(1):78. 22726453 10.1186/1479-5868-9-78 PMC3441783

[22] Cross R, Greaves C, Withall J, Kritz M, Stathi A. A qualitative longitudinal study of motivation in the REtirement in ACTion (REACT) physical activity intervention for older adults with mobility limitations. Int J Behav Nutr Phys Act. 2023;20(1):52. 37101268 10.1186/s12966-023-01434-0 PMC10131311

[23] Palombi T, Chirico A, Cazzolli B, Zacchilli M, Alessandri G, Filosa L, et al. Motivation, psychological needs and physical activity in older adults: a qualitative review. Age Ageing. 2025;54(7):afaf180. 40601367 10.1093/ageing/afaf180 PMC12218189

[24] Bandura A. Self-efficacy: the exercise of control. New York (NY): W.H. Freeman; 1997.

[25] Collado-Mateo D, Lavín-Pérez AM, Peñacoba C, Del Coso J, Leyton-Román M, Luque-Casado A, et al. Key factors associated with adherence to physical exercise in patients with chronic diseases and older adults: an umbrella review. Int J Environ Res Public Health. 2021;18(4):2023. 33669679 10.3390/ijerph18042023 PMC7922504

[26] Xie L, Ma W, Du K, Huang Y, Li A, Wang H, et al. Association between exercise self-efficacy and physical activity in elderly individuals: a systematic review and meta-analysis. Front Psychol. 2025;16:1589076. 10.3389/fpsyg.2025.1525277 PMC12185527 40557371

[27] Camp N, Vagnetti R, Penner S, Ramos C, Hunter K, Hough J, et al. It is not just a matter of motivation: the role of self-control in promoting physical activity in older adults—a Bayesian mediation model. Healthc (Basel). 2024;12(16):1663. 10.3390/healthcare12161663 PMC11353447 39201220

[28] Mikkelsen N, Dall CH, Frederiksen M, Holdgaard A, Rasmusen H, Prescott E. The motivation for physical activity is a predictor of VO2peak and is a useful parameter when determining the need for cardiac rehabilitation in an elderly cardiac population. PLoS ONE. 2022;17(9):e0275091. 36170331 10.1371/journal.pone.0275091 PMC9518852

[29] Martínez-Kercher VM, Watkins JM, Goss JM, Phillips LA, Roy BA, Blades K, et al. Psychological needs, self-efficacy, motivation, and resistance training outcomes in a 16-week barbell training program for adults. Front Psychol. 2024;15:1458421. 39286563 10.3389/fpsyg.2024.1439431 PMC11404365

[30] Rivera-Torres S, Fahey TD, Rivera MA. Adherence to exercise programs in older adults: informative report. Gerontol Geriatr Med. 2019;5:2333721418823604. 30733977 10.1177/2333721418823604 PMC6343518

[31] Picorelli AMA, Pereira LSM, Pereira DS, Felício D, Sherrington C. Adherence to exercise programs for older people is influenced by program characteristics and personal factors: a systematic review. J Physiother. 2014;60(3):151–6. 25092418 10.1016/j.jphys.2014.06.012

[32] Hawley-Hague H, Horne M, Skelton DA, Todd C. Review of how we should define (and measure) adherence in studies examining older adults’ participation in exercise classes. BMJ Open. 2016;6(6):e011560. 27338884 10.1136/bmjopen-2016-011560 PMC4932302

[33] Domingos C, Correia Santos N, Pêgo JM. Association between self-reported and accelerometer-based estimates of physical activity in Portuguese older adults. Sens (Basel). 2021;21(7):2258. 10.3390/s21072258 PMC8038119 33804834

[34] Prince SA, Cardilli L, Reed JL, Saunders TJ, Kite C, Douillette K, et al. A comparison of self-reported and device measured sedentary behaviour in adults: a systematic review and meta-analysis. Int J Behav Nutr Phys Act. 2020;17(1):31. 32131845 10.1186/s12966-020-00938-3 PMC7055033

[35] Garber CE, Blissmer B, Deschenes MR, Franklin BA, Lamonte MJ, Lee IM, et al. Quantity and quality of exercise for developing and maintaining cardiorespiratory, musculoskeletal, and neuromotor fitness in apparently healthy adults. Med Sci Sports Exerc. 2011;43(7):1334–59. 21694556 10.1249/MSS.0b013e318213fefb

[36] Collins LM, Lanza ST. Latent class and latent transition analysis: with applications in the social, behavioral, and health sciences. Hoboken (NJ): Wiley; 2010.

[37] Howard MC, Hoffman ME. Variable-centered, person-centered, and person-specific approaches: where theory meets the method. Organ Res Methods. 2018;21(4):846–76.

[38] Fosstveit SH, Berntsen S, Feron J, Joyce KE, Ivarsson A, Segaert K, et al. HIIT at home: enhancing cardiorespiratory fitness in older adults—a randomized controlled trial. Scand J Med Sci Sports. 2024;34(7):e14702. 38982665 10.1111/sms.14694

[39] Feron J, Rahman F, Fosstveit SH, Joyce KE, Gilani A, Lohne-Seiler H, et al. Cerebral blood flow and arterial transit time responses to exercise training in older adults. NeuroImage. 2024;303:120919. 39505224 10.1016/j.neuroimage.2024.120919

[40] Fernandes EG, Fosstveit SH, Feron J, Rahman F, Lucas SJE, Lohne-Seiler H et al. Effects of increasing fitness through exercise training on language comprehension in monolingual and bilingual older adults: a randomized controlled trial. Aging Neuropsychol Cogn 2024:1–33. 10.1080/13825585.2024.2435914 39693229

[41] Fosstveit SH, Feron J, Lohne-Seiler H, Joyce KE, Segaert K, Lucas SJE, et al. Impact of adherence to six-month home-based HIIT on cardiorespiratory fitness in older adults. BMC Sports Sci Med Rehabil. 2026;18:83. 10.1186/s13102-026-01750-5 PMC13343786 42135794

[42] Markland D, Tobin V. A modification to the Behavioural Regulation in Exercise Questionnaire to include an assessment of amotivation. J Sport Exerc Psychol. 2004;26(2):191–6.

[43] McAuley E. The role of efficacy cognitions in the prediction of exercise behavior in middle-aged adults. J Behav Med. 1992;15(1):65–88. 1583674 10.1007/BF00848378

[44] McAuley E, Szabo A, Gothe N, Olson EA. Self-efficacy: implications for physical activity, function, and functional limitations in older adults. Am J Lifestyle Med. 2011;5(4):361–9. 10.1177/1559827610392704 PMC3864698 24353482

[45] Brown CS, Sloane R, Morey MC. Developing predictors of long-term adherence to exercise among older veterans and spouses. J Appl Gerontol. 2020;39(10):1159–62. 31542972 10.1177/0733464819874954 PMC7083684

[46] Nilsen TS, Scott JM, Michalski M, Capaci C, Thomas S, Herndon JE, et al. Novel methods for reporting of exercise dose and adherence. Med Sci Sports Exerc. 2018;50(6):1134–41. 29315168 10.1249/MSS.0000000000001545 PMC5953772

[47] Borg G. Psychophysical bases of perceived exertion. Med Sci Sports Exerc. 1982;14(5):377–81. 7154893

[48] Sasaki H, Kasagi F, Yamada M, Fujita S. Grip strength predicts cause-specific mortality in middle-aged and elderly persons. Am J Med. 2007;120(4):337–42. 17398228 10.1016/j.amjmed.2006.04.018

[49] Rikli RE, Jones CJ. Development and validation of a functional fitness test for community-residing older adults. J Aging Phys Act. 1999;7(2):129–61.

[50] R Core Team. R: a language and environment for statistical computing. Vienna (Austria): R Foundation for Statistical Computing; 2024.

[51] Akaike H. A new look at the statistical model identification. IEEE Trans Autom Control. 1974;19(6):716–23.

[52] Schwarz G. Estimating the dimension of a model. Ann Stat. 1978;6(2):461–4.

[53] Chaabene H, Prieske O, Herz M, Moran J, Höhne J, Kliegl R, et al. Home-based exercise programmes improve physical fitness of healthy older adults: a PRISMA-compliant systematic review and meta-analysis with relevance for COVID-19. Ageing Res Rev. 2021;67:101265. 33571702 10.1016/j.arr.2021.101265

[54] Liang IJ, Perkin OJ, McGuigan PM, Spellanzon B, Robb M, Liu CY, et al. The effectiveness of unsupervised home-based exercise for improving lower extremity physical function in older adults in Western and Eastern cultures: a systematic review and meta-analysis. BMC Geriatr. 2024;24(1):844. 39354428 10.1186/s12877-024-05393-4 PMC11443890

[55] Geraedts H, Zijlstra A, Bulstra SK, Stevens M, Zijlstra W. Effects of remote feedback in home-based physical activity interventions for older adults: a systematic review. Patient Educ Couns. 2013;91(1):14–24. 23194823 10.1016/j.pec.2012.10.018

[56] Schrack JA, Leroux A, Fleg JL, Zipunnikov V, Simonsick EM, Studenski SA, et al. Using heart rate and accelerometry to define quantity and intensity of physical activity in older adults. J Gerontol Biol Sci Med Sci. 2018;73(5):668–75. 10.1093/gerona/gly029 PMC5905658 29509832

[57] Herrod PJJ, Blackwell JEM, Boereboom CL, Atherton PJ, Williams JP, Lund JN, et al. The time course of physiological adaptations to high-intensity interval training in older adults. Aging Med (Milton). 2020;3(4):245–51. 33392430 10.1002/agm2.12127 PMC7771560

[58] Fyfe JJ, Dalla Via J, Jansons P, Scott D, Daly RM. Feasibility and acceptability of a remotely delivered, home-based, pragmatic resistance exercise snacking intervention in community-dwelling older adults: a pilot randomised controlled trial. BMC Geriatr. 2022;22(1):541. 35751032 10.1186/s12877-022-03207-z PMC9233333

[59] Lang S, McLelland C, MacDonald D, Hamilton DF. Do digital interventions increase adherence to home exercise rehabilitation? A systematic review of randomised controlled trials. Arch Physiother. 2022;12(1):31. 10.1186/s40945-022-00148-z PMC9527092 36184611

[60] Chen Y, Ji H, Shen Y, Liu D. Chronic disease and multimorbidity in the Chinese older adults’ population and their impact on daily living ability: a cross-sectional study of the Chinese Longitudinal Healthy Longevity Survey (CLHLS). Arch Public Health. 2024;82(1):24. 38303089 10.1186/s13690-024-01243-2 PMC10832143

[61] Gilchrist H, Oliveira JS, Kwok WS, Sherrington C, Pinheiro MB, Bauman A, et al. Use of behavior change techniques in physical activity programs and services for older adults: findings from a rapid review. Ann Behav Med. 2024;58(3):216–30. 38300788 10.1093/abm/kaad074 PMC10858305

[62] Meade LB, Bearne LM, Sweeney LH, Alageel SH, Godfrey EL. Behaviour change techniques associated with adherence to prescribed exercise in patients with persistent musculoskeletal pain: systematic review. Br J Health Psychol. 2019;24(1):10–30. 29911311 10.1111/bjhp.12324 PMC6585717

[63] Peiris CL, Gallagher A, Taylor NF, McLean S. Behavior change techniques improve adherence to physical activity recommendations for adults with metabolic syndrome: a systematic review. Patient Prefer Adherence. 2023;17:689–97. 36945683 10.2147/PPA.S393174 PMC10024875

[64] Room J, Hannink E, Dawes H, Barker K. What interventions are used to improve exercise adherence in older people and what behavioural techniques are they based on? A systematic review. BMJ Open. 2017;7(12):e019221. 29247111 10.1136/bmjopen-2017-019221 PMC5736048

[65] Solis-Navarro L, Gismero A, Fernández-Jané C, Torres-Castro R, Solá-Madurell M, Bergé C, et al. Effectiveness of home-based exercise delivered by digital health in older adults: a systematic review and meta-analysis. Age Ageing. 2022;51(11):afac243. 36346736 10.1093/ageing/afac243 PMC9642810

[66] Zhou T, Zhang S, Liu S, Yu J. Digital technology integration in home-based exercise: a systematic review of research evolution, applications, and impact mechanisms. BMC Public Health. 2025;25(1):3214. 41107883 10.1186/s12889-025-24679-9 PMC12534984

[67] Emm-Collison LG, Sebire SJ, Salway R, Thompson JL, Jago R. Multidimensional motivation for exercise: a latent profile and transition analysis. Psychol Sport Exerc. 2020;47:101619. 32127781 10.1016/j.psychsport.2019.101619 PMC7015274

[68] Zhong T, Wang H. Motivation profiles for physical activity among office workers. Front Psychol. 2019;10:2067. 31354582 10.3389/fpsyg.2019.01577 PMC6636603

[69] Woo SH, Seo JP, Kim HR, So WY, Sim YK. Health-promoting behaviors, physical self-efficacy, exercise adherence, and sports commitment among older adults who participate in sports activities. Healthc (Basel). 2024;12(21):2135. 10.3390/healthcare12212135 PMC11545074 39517347

[70] Gillison FB, Rouse P, Standage M, Sebire SJ, Ryan RM. A meta-analysis of techniques to promote motivation for health behaviour change from a self-determination theory perspective. Health Psychol Rev. 2019;13(1):110–30. 30295176 10.1080/17437199.2018.1534071

[71] Ntoumanis N, Ng JYY, Prestwich A, Quested E, Hancox JE, Thøgersen-Ntoumani C, et al. A meta-analysis of self-determination theory-informed intervention studies in the health domain: effects on motivation, health behavior, physical, and psychological health. Health Psychol Rev. 2021;15(2):214–44. 31983293 10.1080/17437199.2020.1718529

[72] Costa-Brito AR, Bovolini A, Rúa-Alonso M, Vaz C, Ortega-Morán JF, Blas Pagador J, et al. Home-based exercise interventions delivered by technology in older adults: a scoping review of technological tools usage. Int J Med Inf. 2024;181:105287. 10.1016/j.ijmedinf.2023.105287 37972483

[73] Di Pumpo M, Miatton A, Riccardi MT, Graps EA, Baldo V, Buja A, et al. Digital health interventions to promote physical activity in community-dwelling older adults: a systematic review and semiquantitative analysis. Int J Public Health. 2025;69:1607720. 39830161 10.3389/ijph.2024.1607720 PMC11738617

[74] Ratz T, Pischke CR, Voelcker-Rehage C, Lippke S. Distinct physical activity and sedentary behavior trajectories in older adults during participation in a physical activity intervention: a latent class growth analysis. Eur Rev Aging Phys Act. 2022;19(1):2. 34986783 10.1186/s11556-021-00281-x PMC8903622

[75] Hagerman CJ, Miller NA, Butryn ML. Latent profile analysis of physical activity motivation during behavioral weight loss treatment. Psychol Sport Exerc. 2023;66:102376. 37383031 10.1016/j.psychsport.2022.102376 PMC10299802

